# Stroke subtypes, risk factors and mortality rate in northwest of Iran

**Published:** 2017-07-06

**Authors:** Mehdi Farhoudi, Kaveh Mehrvar, Homayoun Sadeghi-Bazargani, Mazyar Hashemilar, Manouchehr Seyedi-Vafaee, Elyar Sadeghi-Hokmabad, Reza Rikhtegar, Babak Saber-Maroof, Mohammad Abutalebi, Mahsa Rezaei, Sahar Vaferi, Alireza Aghili, Omid Ebrahimi

**Affiliations:** 1 Neurosciences Research Center, Tabriz University of Medical Sciences, Tabriz, Iran; 2 Iranian Social Security Organization, Tabriz University of Medical Sciences, Tabriz, Iran; 3 Department of Nuclear Medicine, University of Southern Denmark, Odense, Denmark

**Keywords:** Stroke, Epidemiology, Risk Factors, Iran

## Abstract

**Background:** Stroke is the second most common cause of death and first cause of disability in adults in the world. About 80% of all stroke deaths occur in developing countries. So far, the data on stroke epidemiology have been limited in Iran. Therefore, this study was focused on stroke demographic data, risk factors, types and mortality.

**Methods:** A retrospective study was done in two university tertiary referral hospitals in Tabriz, northwest of Iran, from March 2008 to April 2013. Patients diagnosed with stroke were enrolled in the study. Demographic data, stroke subtypes, duration of hospitalization, stroke risk factors and hospital mortality rate were recorded for all the patients.

**Results:** A total number of 5355 patients were evaluated in the present study. Mean age of the patients was 67.5 ± 13.8 years, and 50.6% were men. Final diagnosis of ischemic stroke was made in 76.5% of the patients, intra-cerebral hemorrhage (ICH) with or without intra-ventricular hemorrhage (IVH) in 14.3% and subarachnoid hemorrhage (SAH) in 9.2%. Stroke risk factors among the patients were hypertension in 68.8% of the patients, diabetes mellitus (DM) in 23.9%, smoking in 12.6% and ischemic heart diseases (IHD) in 17.1%. Mean hospital stay was 17.3 days. Overall, the in-hospital mortality was 20.5%.

**Conclusion:** Compared to other studies, duration of hospital stay was longer and mortality rate was higher in this study. Hypertension was the most common risk factor and cardiac risk factors and DM had relatively lower rate in comparison to other studies. Because of insufficient data on the epidemiology, patterns, and risk factors of stroke in Iran, there is a necessity to develop and implement a national registry system.

## Introduction

Stroke is a serious problem world-wide especially in Asia with higher mortality than Europe or North America.^1^ It is the most important cause of disability and the second cause of death worldwide. Only one-third of all strokes occur in the developed countries. In the developing countries stroke is a major health issue despite being preventable.^2^ It is estimated that about 5.7 million deaths in 2005 occurred that most of these deaths (87%) were in low-income and middle-income countries. Nowadays the incidence of stroke in low- to middle-income countries is higher than in high-income countries. Moreover in low to middle-income countries, there are greater mortality rate and a younger age of stroke onset, factors that raise the burden of stroke burden.^3^ Stroke constitutes a major global challenge for health policy and healthcare economics. Reducing stroke burden requires extensive knowledge of risk factors and, if applicable, preventive control. In last classification of countries by World Bank, Iran was qualified as a middle-income country,^4^ and the stroke prevalence in Iran is significantly higher than the developed countries especially for stroke in young adults.^5^^,^^6^ In comparison with high-income countries, stroke in young adult is more common in Iran and mortality rate is higher.^7^ Fortunately, stroke is a preventable disease and for this purpose, knowledge of risk factors and epidemiology of it within a certain country is an essential step.^8^ This study was conducted in order to collect the epidemiological data in patients diagnosed with stroke in order to help the healthcare providers manage stroke more effectively in Iran.

## Materials and Methods

This was a retrospective hospital-based, longitudinal study which was performed at the Imam Reza and Razi Hospitals, two major tertiary referral centers in northwest of Iran affiliated to Tabriz University of Medical Sciences, Tabriz, Iran. All patients with the diagnosis of stroke from March 2008 to April 2013 were enrolled. Stroke patients from any subtypes (ischemic, hemorrhagic) were selected based on the patients’ data in hospital documents and by using the International Classification of Diseases, 10^th^ edition (ICD-10). All available data including age, gender, duration of hospital stay, discharge state, mortality, risk factors and paraclinical data were sought in especially-designed data matrix. Risk factors for each subtype of stroke were recorded separately. The patients were followed up during hospital stay. Chi-square test and Student’s t-test were used to analyze the data. For hospital mortality outcome, a multivariate logistic regression analysis was built with sex and age groups as covariates. A P-value less than 0.05 was considered significant. For statistical analysis, SPSS software (version 18, SPSS Inc., Chicago, IL, USA) was used.

## Results

**Figure 1:**
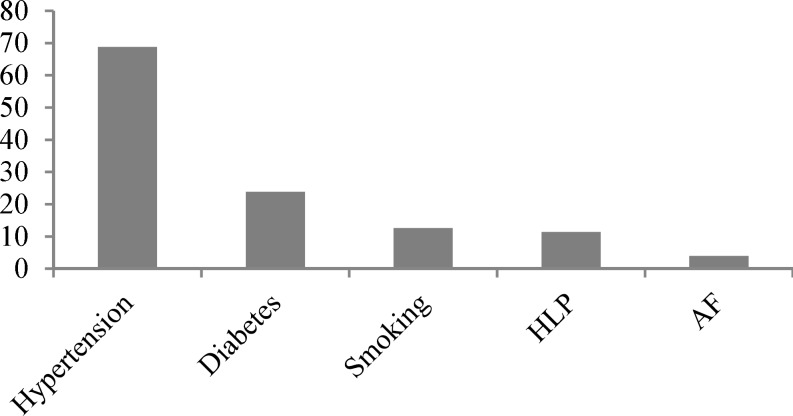
Frequency of risk Factors

HLP: Hyperlipidemia; AF: Atrial fibrillation

Medical records from 5355 patients consisting of 2708 (50.6%) men and 2647 (49.4%) women were reviewed. Table 1 illustrates general feature of the study. The mean age of patients was 67.6 ± 13.8 years. Among the patients 7 (0.1%) were in pediatric age group (< 15 years), 414 (7.7%) were young adults (15-45 years), 1587 (29.6%) were middle aged (45-65 years) and 3374 (63%) were older adults (< 65 years). In this study stroke subtypes included ischemic stroke 4096 (76.5%), intra-cerebral hemorrhage (ICH) with/without intra-ventricular hemorrhage (IVH) 764 (14.3%) and subarachnoid hemorrhage (SAH) 495 (9.2%). Conventional risk factors were as follows: hypertension that was the most frequent and recorded in 3686 (68.8%) of patients, diabetes mellitus (DM) in 1278 (23.9%), smoking in 673 (12.6%), hyperlipidemia (HLP) in 613 (11.4%) atrial fibrillation (AF) in 215 (4.0%), familial history of stroke in 69 (1.5%) and congestive heart failure in 74 (1.4%) (Figure 1).

**Figure 2:**
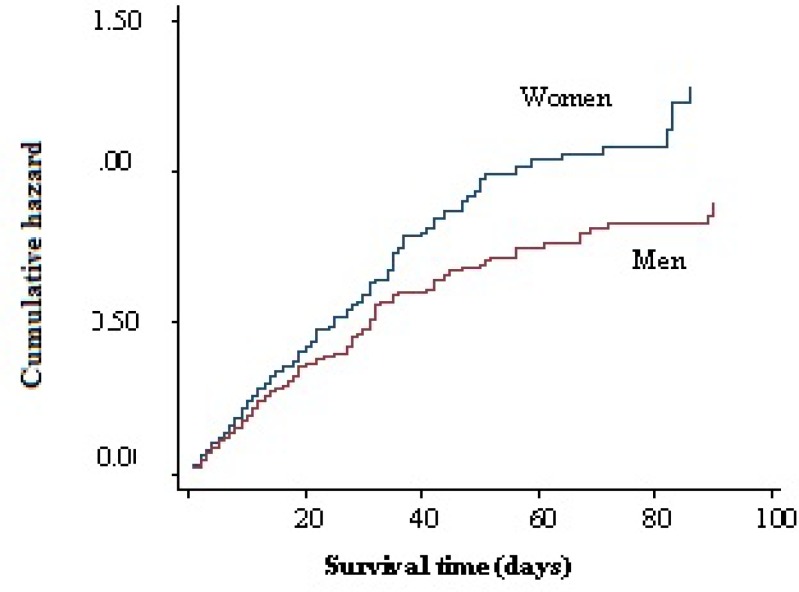
Correlation between gender and mortality

**Figure 3:**
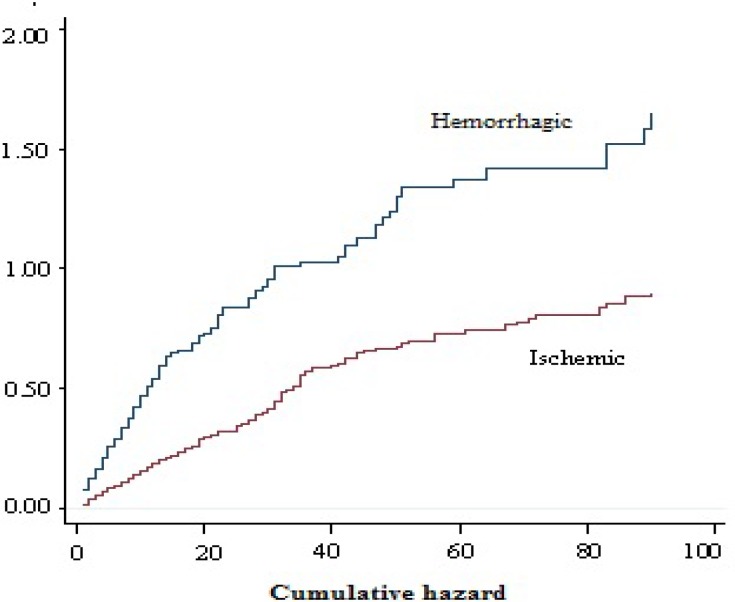
Correlation between type of stroke and mortality

**Figure 4:**
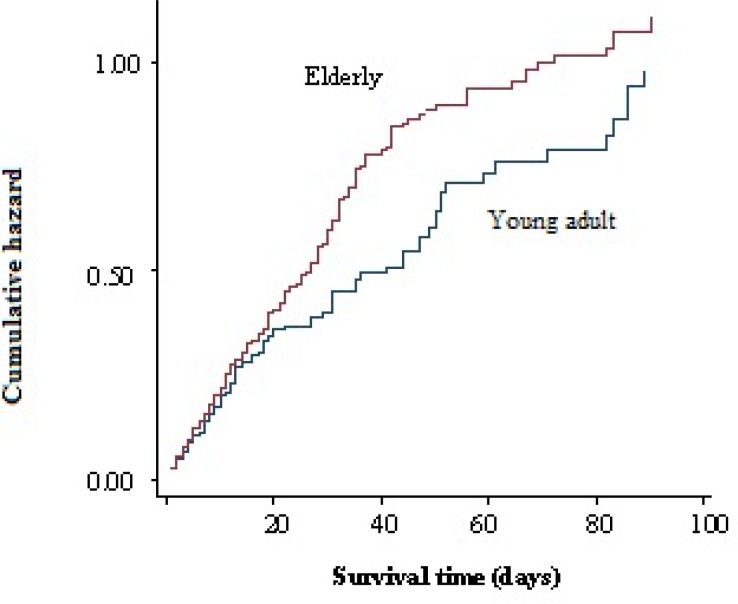
Correlation between age and mortality

In young adults hypertension was less common (29.9%) but smoking was more frequent (15.9%). Hypertension was more common in ischemic stroke patients compared with ICH or SAH patients. Mean hospital stay was 17.3 days which was longer in mortality cases (25.8 days). A total of 1099 (20.5%) patients died during the hospitalization of which 577 (52.5%) were men and 522 (47.5%) women (Figure 2). Mortality rate in hemorrhagic patients was higher than ischemic ones (Figure 3). The mean age in deceased patients was higher than the average age of all patients (69.9 years and 67.6 years, respectively; P < 0.001) and the mortality rate in group aged > 65 years was more than twice of the rest (69.9% vs. 33.1%) (P < 0.001) (Figure 4).

**Table 1:** Baseline characteristics of hospital admitted stroke patients in northwest of Iran (2008-2013)

**Characteristics**	**Men**	**Women**	**Total (%)**
	**(n = 2708)**	**(n = 2647)**	**(n = 5355)**
Age (year) (mean ± SD)	65.2 ± 14.6	64.2 ± 15.0	67.5 ± 13.8
Risk factors history [n (%)]			
Hypertension	1691 (62.4)	1995 (75.4)	68.8
DM	558 (20.6)	720 (27.2)	23.9
HLP	242 (8.9)	371 (14.0)	11.4
AF	127 (3.2)	88 (4.8)	4.0
TIA	66 (2.4)	33 (1.2)	2.1
Smoking	573 (21.2)	100 (3.8)	12.6
Ischemic heart disease	460 (17.0)	456 (17.2)	17.1
Familial history of stroke	40 (1.5)	39 (1.5)	1.5
Types of stroke [n (%)]			
Ischemic	2083 (76.9)	2013 (76.0)	76.5
ICH	398 (14.6)	366 (13.8)	14.3
SAH	227 (8.5)	268 (10.2)	9.2
Mortality [n (%)]	577 (21.3)	522 (19.7)	20.5

SD: Standard deviation; TIA: Transient ischemic attack; ICH: Intra-cerebral hemorrhage; SAH: Subarachnoid hemorrhage; DM: Diabetes mellitus; HLP: Hyperlipidemia; AF: Atrial fibrillation

The important results of this study have been summarized in table 1.

## Discussion

In this study the prevalence of stroke among men and women were relatively equal. The mean age of patients was as the same as other studies in Iran^8^ and India^9^ and Saudi Arabia^10^ but slightly higher than Saudi Arabia.^10^ The lower mean age in Saudi Arabia might be due to predominance of young age group in that country. Likewise, in developed countries ischemic stroke represents the majority of stroke subtypes, followed by ICH and SAH.^3^^,^^11^^,^^12^

The stroke patients age categories in the study with respect to the population in each age category in East Azerbaijan province in 2010 based on national census is shown in table 2. It could be implied that the stroke risk increases with aging and reach the maximum in 9^th^ decade of life in this population that is similar to other studies.^13^

**Table 2:** Age distribution of the stroke patients admitted to the Imam Reza and Razi hospitals with respect to the population in Tabriz

**Age (year)**	**Men [n (%)]**		**Women [n (%)]**		**Total [n (%)]**		**Ratio** ***
	**Admission** *	**Population** **	**Admission**	**Population**	**Admission**	**Population**	
10-19	3 (0.10)	291073 (15.40)	3 (0.10)	567741 (30.80)	6 (0.10)	858814 (23.10)	0.004
20-29	37 (1.36)	405376 (21.50)	43 (1.62)	394678 (21.40)	80 (1.49)	800054 (21.50)	0.069
30-39	90 (3.30)	323096 (17.20)	96 (3.62)	318219 (17.30)	186 (3.47)	641315 (17.20)	0.200
40-49	206 (7.60)	232682 (12.40)	225 (8.53)	228377 (12.40)	431 (8.04)	461059 (12.40)	0.640
50-59	415 (15.40)	161438 (8.57)	449 (17.00)	166225 (9.02)	864 (16.20)	327663 (8.79)	1.830
60-69	657 (24.40)	88348 (4.69)	528 (19.90)	95881 (5.20)	1185 (22.20)	184229 (4.94)	4.490
70-79	832 (30.70)	63828 (3.39)	866 (32.70)	62214 (3.37)	1698 (31.70)	126042 (3.38)	9.370
80-89	445 (16.40)	25288 (1.34)	416 (15.70)	25168 (1.36)	861 (16.10)	50456 (1.35)	11.900
≥ 90	23 (0.84)	1604 (0.08)	21 (0.79)	2012 (0.10)	44 (0.88)	3606 (0.10)	8.800
Total	2708	1882031	2647	1842589	5355	3724620	0.140

* Admitted stroke patients to Imam Reza and Razi hospitals,

** East Azerbaijan population,

*** Calculated by dividing the percent admitted to the hospital to the percent population in each category

This pattern of highly prevalent risk factors is similar to the Arab countries neighboring the Persian Gulf except for a relatively low incidence of DM and cardiac problems in this study.^9^^,^^10^^-^^14^

This study revealed that hypertension was the most frequent risk factor in all subtypes of stroke across all age groups that was similar to all other epidemiologic studies. Moreover, the risk of hypertension was similar among all the patients with stroke due to either large vessel disease or lacunar infarct, like the Oxfordshire project.^15^

DM is a strong risk factor for stroke,^16^ and its prevalence is increasing.^17^ Persons with DM have an increased susceptibility to atherosclerosis and atherogenic risk factors, notably hypertension, obesity, and abnormal blood lipids.^18^ It is still unclear whether stroke subtype, severity, and prognosis are different in diabetic and nondiabetic patients.^16^ In this study, DM was the second most important risk factor but with lower incidence in comparison with other studies.^6^ Low incidence of DM as a risk factor probably is due to low incidence of this disease in northwest of Iran in comparison with other places or unawareness of people about their disease.

Cardiac disorders are modifiable risk factors for stroke. Cardioembolic stroke accounts for 14%-30% of all cerebral infarctions.^19^ In one study 14% of stroke patients were diagnosed with cardiogenic stroke.^20^ Unlike other studies in developed and developing countries cardiac problems had a small role as a risk factor in this study. For example, AF was detected in only 4.0% of our patients in comparison with 8.6% of patients in another study.^6^ This is maybe due to incomplete data recording and in-hospital cardiac survey in our centers. 

The other important risk factors for ischemic stroke and transient ischemic attack (TIA) are smoking and HLP which are considered as two modifiable risks. The well-known association between smoking and ischemic stroke can be attributed to large-vessel atherosclerosis with stenosis.^21^^,^^22^

According to the previous studies, cigarette smoking was correlated with atherosclerotic and cardioembolic types of ischemic stroke.^23^ In this study smoking with 12.6% and HLP with 11.4% prevalence, were important risk factors in both ischemic and hemorrhagic strokes.

Stroke incidence increases with a family history of stroke.^6^ This fact could be due to a familial association existing with other risk factors for stroke (cholesterol, hyperfibrinogenemia, hypertension, diabetes and etc.),^18^ genetic tendency for stroke, a genetic determination of other stroke risk factors, and a common familial exposure to environmental or lifestyle risks,^17^ or due to independent factors.^18^

In this study the incidence of family history of stroke was 1.5%, lower in comparison with other studies,^24^ and it might be duo to incompetent medical recording in our hospitals. Mean hospital stay was longer than another study in southern Iran,^7^ because our centers are referral for 4-5 provinces of Iran and some neighbor countries with many patients with poor prognosis. Our in-hospital mortality rate (20.5%) was higher in comparison with some developed countries (17.5%),^25^ and equal to (20.0%) or lower (24.6%) than other studies in Iran.^5^^,^^7^

The 30-day mortality rate of stroke patients in North Africa and Arab Middle-Eastern countries, which are socioeconomically similar to Iran, is lower and reported between 10% and 17.3%.^26^ On the other hand unlike most other studies, in this study mortality rate was higher in men than women that can be due to Iranian culture and life style, as women are less involved in stressful situations and consume much less alcohol and cigarettes.

These results can be due to some factors such as absence of primary and secondary stroke units and consequently low rate of thrombolysis and thrombectomy that can influence stroke prognosis.^27^ Moreover, incomplete stroke registry system during this study period and low stroke awareness among Iranian general population,^28^ that causes the late referral of stroke patients and leads to increased mortality, are other causes.

## Conclusion

xDuration of hospital stay was longer and mortality rate was higher in Northwest of Iran than other countries. Among risk factors, cardiac risk factors and DM had lower rate in comparison to other studies probably due to under diagnosis. Generally, the data on the epidemiology of stroke and its pattern and risk factors is scarce in Iran. In order to overcome this incompetency and improve the data recording and outcome of stroke patients, we need to develop systematic recording and registries and provide stroke units.

## Notes:

**How to cite this article:** Farhoudi M, Mehrvar K, Sadeghi-Bazargani H, Hashemilar M, Seyedi-Vafaee M, Sadeghi-Hokmabad E, et al. Stroke subtypes, risk factors and mortality rate in northwest of Iran. Iran J Neurol 2017; 16(3): 112-7.
